# An efficient optic cup segmentation method decreasing the influences of blood vessels

**DOI:** 10.1186/s12938-018-0560-y

**Published:** 2018-09-26

**Authors:** Chunlan Yang, Min Lu, Yanhua Duan, Bing Liu

**Affiliations:** 1 College of Life Science and Bioengineering, Beijing University of Technology, Beijing, 100124 China; 2 Department of Ophthalmology, Hospital of Beijing University of Technology, Beijing, 100124 China

**Keywords:** Optic cup, Digital fundus image, Segmentation, Blood vessel, BSCB model

## Abstract

**Background:**

Optic cup is an important structure in ophthalmologic diagnosis such as glaucoma. Automatic optic cup segmentation is also a key issue in computer aided diagnosis based on digital fundus image. However, current methods didn’t effectively solve the problem of edge blurring caused by blood vessels around the optic cup.

**Methods:**

In this study, an improved Bertalmio–Sapiro–Caselles–Ballester (BSCB) model was proposed to eliminate the noising induced by blood vessel. First, morphological operations were performed to get the enhanced green channel image. Then blood vessels were extracted and filled by improved BSCB model. Finally, Local Chart-Vest model was used to segment the optic cup. A total of 94 samples which included 32 glaucoma fundus images and 62 normal fundus images were experimented.

**Results:**

The evaluation parameters of F-score and the boundary distance achieved by the proposed method against the results from experts were 0.7955 ± 0.0724 and 11.42 ± 3.61, respectively. Average vertical optic cup-to-disc ratio values of the normal and glaucoma samples achieved by the proposed method were 0.4369 ± 0.1193 and 0.7156 ± 0.0698, which were also close to those by experts. In addition, 39 glaucoma images from the public dataset RIM-ONE were also used for methodology evaluation.

**Conclusions:**

The results showed that our proposed method could overcome the influence of blood vessels in some degree and was competitive to other current optic cup segmentation algorithms. This novel methodology will be expected to use in clinic in the field of glaucoma early detection.

## Background

Optic cup is an important retinal structure in ophthalmologic diagnosis such as glaucoma [1, 2]. Glaucoma is the second leading disease to blindness worldwide [3]. Given the lack of visual symptoms in the early stages, several studies showed that more than 90% of the patients were unaware of this disease until it has developed into the severe stages [4–6]. Since it is time cost and the precision of diagnosis by manual is limited, then the automatic technique for disease detection such as computer aided diagnosis (CAD) system is strongly needed [7].

With the development of computer science, medical image processing technique has been successfully applied to clinical diagnosis and treatment. Currently, digital fundus image has been widely used in many hospitals. Thus, it is possible to develop the CAD system used for ophthalmologic diagnosis based on fundus image processing technique [8–11].

The OD includes two distinct parts, namely, a circumjacent zone called the rim and a central bright region called the optic cup [12]. The optic cup can be divided into nasal and temporal regions. The former is generally occluded by the main blood vessels. Physiologically, the loss in optic nerve fibers (ONF) leads to the enlargement of optic cup which is called large cupping, and atrophy of neuroretinal rim which is called rim loss. Large cupping and rim loss are two important indicators of glaucoma. They could be detected by measuring the vertical optic cup-to disc ratio (CDR) [13]. Once more ONF disappear, the optic cup will become larger with respect to the optic disc, which corresponds to CDR increasing. In generally, an abnormal vertical CDR indicate a high risk of glaucoma. Therefore, automatic segmentation of the optic cup is crucial for CAD system for glaucoma.

To date, there are few studies on optic cup segmentation algorithms, which include thresholding-based methods [14–16], region growing [17, 18], model-based methods (active contour models or snakes [19, 20], level sets [21, 22], and elliptical shape model [23]), anatomical evidence-based methods [24], and superpixel classification [25–27]. Optic cup segmentation is challenged because the intensity has a sudden change in areas which blood vessels pass across the cup-disc boundary. However, most studies haven’t solved this problem effectively. Thus, inpainting of the blood vessels is an important step in optic cup segmentation.

This study aims to develop an efficient method to overcome the aforementioned problem in optic cup segmentation. Morphological operations were first performed to get the enhanced green channel image. Then, blood vessels were extracted and filled by using an improved Bertalmio–Sapiro–Caselles–Ballester (BSCB) model. Finally, local chart-vest (LCV) model was used to segment the optic cup.

The remainder of this paper is organized as follows: first, we introduced the image data used in this study and presented the proposed methodology, an efficient optic cup segmentation method decreasing the influences of blood vessels. The experimental results are then provided, followed by discussions and conclusions.

## Methods

### Data acquisition

Digital fundus images were acquired from local ophthalmologic hospital. The 24-bit color images were captured using digital fundus camera (Canon CR-DGi) with the array size of 1440 × 960 pixels. The photographic angle of the fundus camera was set to 60°, and the optic disc was adjusted at image center. The average optic cup boundary identified by two experts was taken as the ground truth for the following evaluation. All the experimented 94 images were acquired by the same instrument and the subjects were collected randomly to take the ocular disease screening. A total of 94 images including 32 patients with glaucoma (18 male, 14 female) and 62 healthy subjects (34 male, 28 female) were included in our experiment. For the 32 patients with glaucoma, the ages were ranged from 35 to 58 years old (45.23 ± 3.31 years). None of the left 62 normal subjects had history of hypertension, nor cardiovascular disease and diabetes. Their ages were ranged from 31 to 63 years old (43.51 ± 5.27 years). In addition, 39 glaucoma images from the public dataset RIM-ONE (An Open Retinal Image Database for Optic Nerve Evaluation) were also used for evaluation [28].

### Optic cup segmentation

The edge of optic cup is more difficult to identify compared with that of optic disc, primarily because the image is blurred where the blood vessels pass across the optic cup. After preprocessing such as image enhancement, blood vessels were extracted and inpainting. Then the optic cup was segmented by using LCV model.

#### Preprocessing

**Fig. 1:**
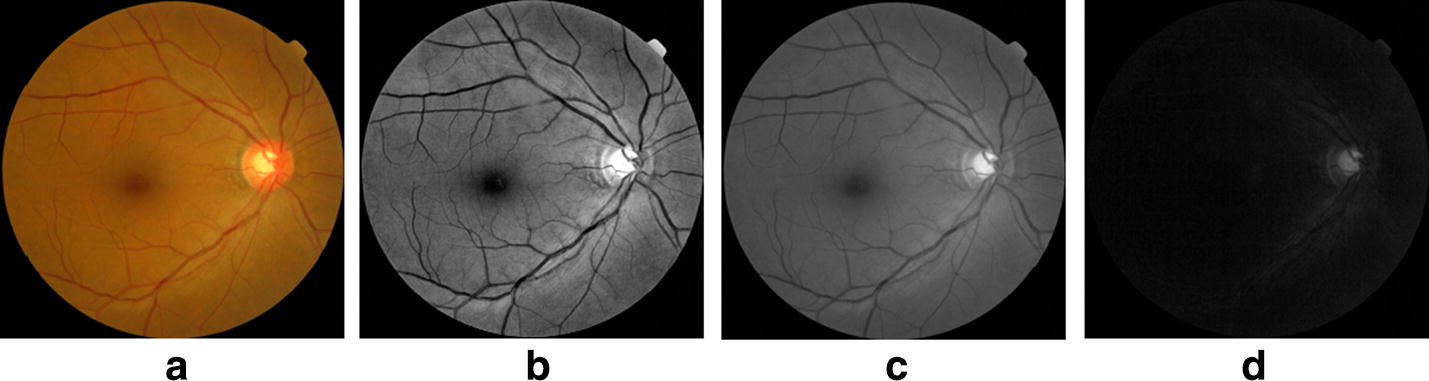
Digital fundus images with different color channels. **a** Original image, **b** red channel image, **c** green channel image, **d** blue channel image

Among the image components of color which are red (R), green (G) and blue (B), the G channel shows the optimal image contrast for the optic cup (see Fig. 1). Therefore, the G channel image *U*_*G*_ was selected for the subsequent processing.

In the proposed algorithm, both top-hat and bottom-hat transformations were applied to enhance the image contrast [27]. Top-hat transformation refers to the subtraction of the opening operation result from the image itself. By contrast, bottom-hat transformation refers to the subtraction of the image from the result of closing operation. Both top-hat and bottom-hat transformations are based on a predefined neighborhood or structuring element (*SE*). The above two transformations are illustrated as Eqs. (1) and (2), respectively.

1 $$ T_{hat} \left( {U_{G} } \right) = U_{G} - \left( {U_{G} \circ SE} \right) $$

2 $$ B_{hat} \left( {U_{G} } \right) = \left( {U_{G} \cdot SE} \right) - U_{G} $$

**Fig. 2:**
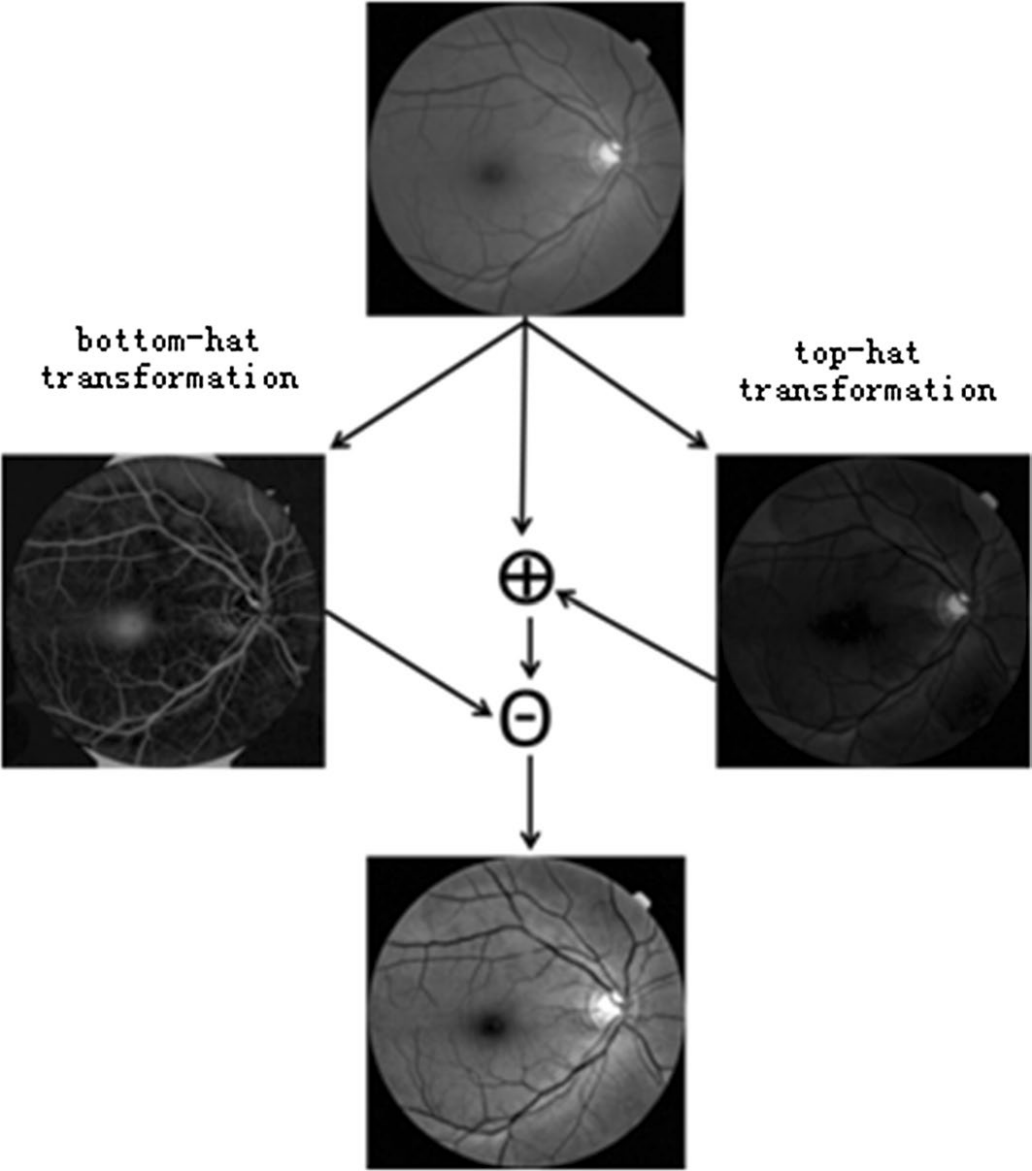
Flowchart of image contrast enhancement by top-hat and bottom-hat transforms. The top is the original image and the bottom is the result

Here, a structuring element with a size of 5 × 5 pixels was used (more details of size selection were provided in “Results” section). The above two transformations could be represented as Eq. (3), the boundary of optic cup become clearer after the operations (see Fig. 2).

3 $$ U = \left( {U_{G} + T_{hat} \left( {U_{G} } \right)} \right) - B_{hat} \left( {U_{G} } \right) $$

#### Blood vessel extraction

Since the intensities of blood vessels were lower than those of background and other structures, the intensities of blood vessels will become higher after median filtering. Therefore, the blood vessels could be possibly identified based on the intensity difference. In order to capture the change of image intensity, the contrast enhanced image *U* was subtracted from the median filtered image *U*_*med*_. Then the intensity differential image could be acquired which was represented by Eq. (4).

4 $$ U_{sub} = U_{med} (i,\;j) - U(i,\;j) $$

**Fig. 3:**
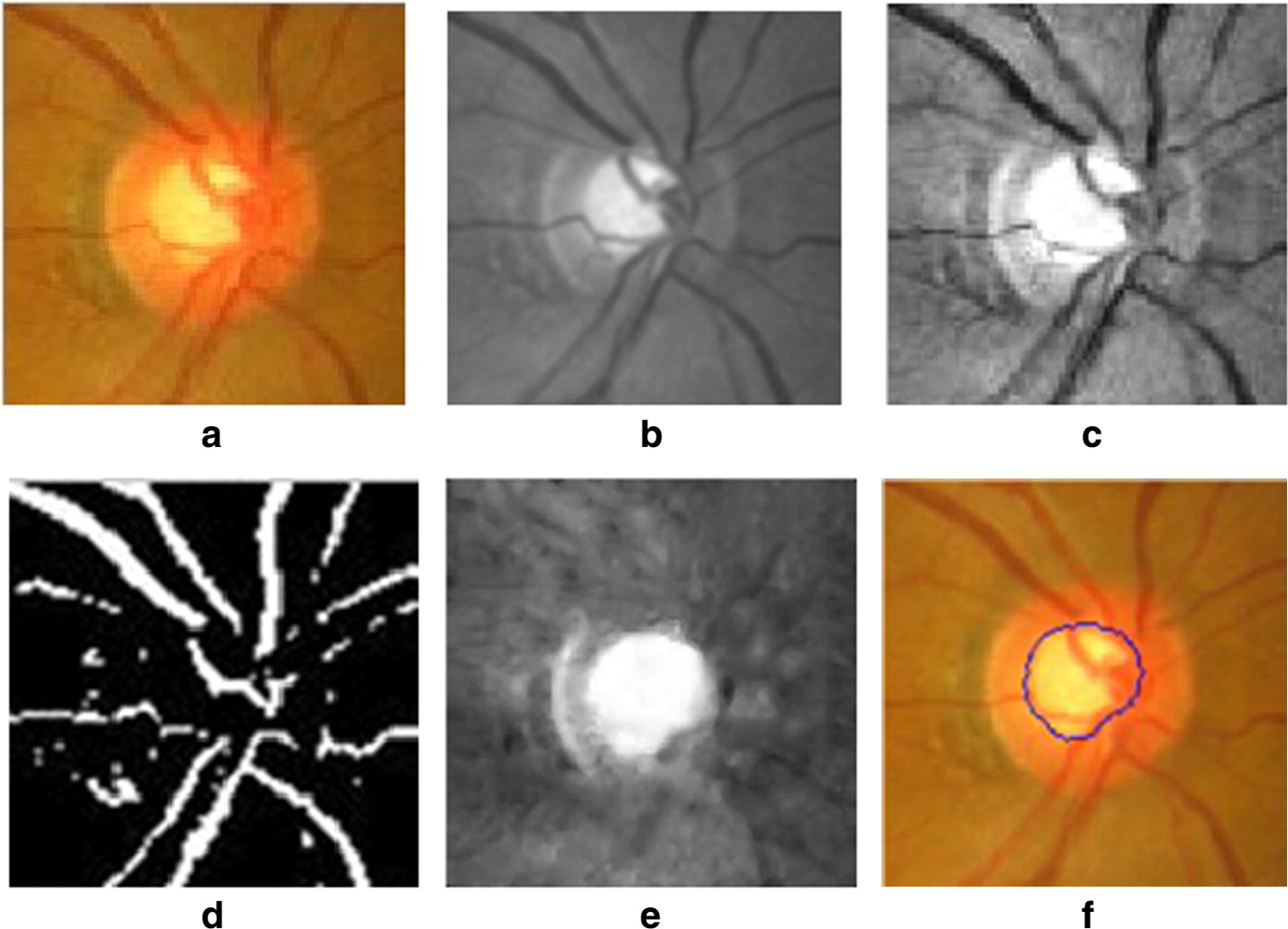
Key steps in proposed optic cup segmentation. **a** Original image, **b** G channel image, **c** enhanced G channel image, **d** blood vessel identification, **e** result of blood vessel inpainting, **f** optic cup identification

The binary image *U*_*D*_ was generated according to the value of *U*_*sub*_ [29]. Only the intensities of pixels with corresponding *U*_*sub*_ > 0 were set to 1 according to Eq. (5). These pixels were considered belonging to blood vessels (see Fig. 3d).

5 $$ U_{D} (i,\;j) = \left\{ {\begin{array}{*{20}ll} {1,} \quad {{\text{if }}U_{sub} > 0} \\ {0,} \quad {\text{otherwise}} \\ \end{array} } \right..$$

In this study, the size of median filtering was set to 9 × 9 pixels (more details of size selection were provided in “Results” section).

#### Blood vessels inpainting

Bertalmio et al. [30] proposed the novel BSCB model for image inpainting in 2000 based on theory of partial differential equation (PDE). The BSCB model uses Laplace operator to measure the neighborhood information for image inpainting. It smoothly propagates the information to the same region along the direction of isophote. At the same time, an anisotropic diffusion function is adopted to prevent the prolongation lines from crossing one to another. Generally, the model comprises two steps: inpainting and diffusion. In this study, the improved BSCB model was developed, that is, the neighborhood intensities were used as the propagation information, instead of using only one single pixel.

After the above processing, the intensity will become more uniform within the area of optic cup. The influence caused by the blood vessels will be decreased. The following were the detailed descriptions about the processing procedure.

Assuming Ω is the region to be inpainted and ∂*D* is the boundary of Ω. The BSCB model can be described by Eqs. of (6) and (7) [31, 32].

6 $$ \frac{\partial U}{\partial t} = \nabla L \cdot \vec{T} $$

7 $$ \frac{\partial U}{\partial t} = g_{\varepsilon } k\left| {\nabla U} \right|. $$

Equation (6) represents inpainting, where ∇*L* is the propagation information and $\vec{T}$ is the isophote direction. Equation (7) is used for diffusion, where *k* is the Euclidean curvature of the isophote and Ω^*ɛ*^ is the dilation of Ω with a balling radius of *ε*, and *g*_*ɛ*_ is a smoothing function of Ω^*ɛ*^.

To be easier understanding, Eqs. of (6) and (7) can be comprehensively described as follows.

8 $$ U^{n + 1} \left( {i,\;j} \right) = U^{n} \left( {i,\;j} \right) + \Delta tU_{\tau }^{n} \left( {i,\;j} \right),\quad \forall \left( {i,\;j} \right) \in\Omega , $$

9 $$ U^{n + 1} \left( {i,\;j} \right) = U^{n} \left( {i,\;j} \right) + \Delta tg_{\varepsilon } \left( {i,\;j} \right)k\left( {i,\;j,\;n} \right)\left| {\nabla U\left( {i,\;j,\;n} \right)} \right|, \quad \forall \left( {i,\;j} \right) \in\Omega ^{\varepsilon } , $$

Here *U*^*n*+1^(*i*, *j*) is the value of pixel intensity located at (*i*, *j*) in the *n*-*th* iteration image which $U_{\tau }^{n} (i,\;j) = \nabla L \cdot \vec{T}$. Notably, *U*^0^(*i*, *j*) = *U*(*i*, *j*) and $\mathop {\lim }\limits_{n \to \infty } U^{n}\left( {i,\;j} \right)\, = \,U_{r} \left({i,\; j} \right),$ where *U*^0^(*i*, *j*) is the input image, *U*_*r*_(*i*, *j*) is the output of the algorithm, and Δ*t* is the improvement rate.

In the traditional BSCB model, propagation information *L*^*n*^(*i*, *j*) is substituted by the discrete Laplace operator, which is shown in Eq. (10).

10 $$ L^{n} \left( {i,\;j} \right) = u_{xx}^{n} \left( {i,\;j} \right)u_{yy}^{n} \left( {i,\;j} \right). $$

Although texture could be the transfer information for image inpainting, it is not necessary in optic cup segmentation. In contrast, local image information is more effective. Therefore, using neighborhood mean value as transfer information may eliminate the influence of noise. According to the above deduction, $u_{xx}^{n} \left( {i,\;j} \right)$ and $u_{yy}^{n} \left( {i,\;j} \right)$ were replaced by $\overline{{u_{xx}^{n} }} \left( {i,\;j} \right)$ and $\overline{{u_{yy}^{n} }} \left( {i,\;j} \right)$ in the following vascular inpainting, represented by Eqs. (11) and (12) respectively. In this study, the size of neighborhood was set to 3 × 3 pixels.

11 $$ \overline{{u_{xx}^{n} }} \left( {i,\;j} \right) = \frac{1}{9}\sum\limits_{m = i - 1}^{i + 1} {\sum\limits_{n = j - 1}^{j + 1} {u_{xx}^{n} } } \left( {m,\;n} \right) $$

12 $$ \overline{{u_{yy}^{n} }} \left( {i,\;j} \right) = \frac{1}{9}\sum\limits_{m = i - 1}^{i + 1} {\sum\limits_{n = j - 1}^{j + 1} {u_{yy}^{n} } } \left( {m,\;n} \right) $$

#### Optic cup boundary identification

Wang et al. [33] proposed a LCV model which included local statistical information in level set based segmentation framework. In the algorithm, extended structure tensor (EST) was combined which intensity inhomogeneity could be decreased effectively. In this study, the above-mentioned LCV model was used for the following optic cup segmentation.

First, the centroid (x_*c*_, y_*c*_) was selected from the region with relative high intensity, which was acquired according to the following criteria represented as Eq. (13)

13 $$ {\text{x}}_{c} = \frac{1}{N}\sum\limits_{i = 1}^{N} {x_{i} } ,\quad {\text{y}}_{c} = \frac{1}{N}\sum\limits_{i = 1}^{N} {y_{i} } $$

Here N is the total number of pixels within the target region.

In this step, two parameters need dynamic adjusted in the LCV model, i.e., *α* and *μ*. Commonly, *α* was set to 0.1 or 1, depending on whether the image has intensity inhomogeneity. For *μ*, two corresponding values are adopted: 0.01 × 255^2^ and 0.1 × 255^2^. If several targets need to be detected, *μ* should be small, and vice versa. Because the optic cup area has intensity inhomogeneity and only the optic cup be the target, the values of *α* and *μ* were then set to 0.1 and 0.1 × 255^2^ respectively.

### Evaluation

The experimental results were evaluated by three statistical criteria, namely, F-score (area-based), distance (curve-based) and vertical CDR, which were explained in “F-score (F)”, “Distance (D)”, and “Vertical CDR” sections.

Besides the proposed algorithm, the experiments were also performed by two other known methods, proposed by Joshi et al. [17] and Liu et al. [21]. There are two reasons why the above methods were selected to be compared. First, since the optic cup segmentation could be classified into framework of region based, edge based and hybrid, the proposed algorithm and another two selected methods all belong to region based method. Second, the proposed method aimed at decreasing the influence of blood vessels, which was similar to the other selected methods putting forward to the solutions.

In details, Joshi et al. [17] imposed the expected symmetry of optic cup region by setting threshold to recover the under-segmentation areas located in the blood vessels. Liu et al. [21] used ellipse to redraw the optic cup boundary after employing a combinative algorithm with level set and threshold setting, which was to weaken the noising by blood vessels.

For each method, the evaluation of F-score and distance were compared. The vertical CDR values were presented against manual segmentation results achieved by experts.

#### F-score (F)

The pixel-wise precision and recall values were computed to assess the overlap area between the computed region and the ground truth. These values were defined as Eqs. (14) and (15),

14 $$ precision = \frac{TP}{TP + FP} $$

15 $$ recall = \frac{TP}{TP + FN} $$

where *TP*, *FP*, and *FN* represented the number of true positive, false positive, and false negative pixels respectively. The harmonic mean of the precision and recall values, called F-score (F), was computed to better appreciate the results. The F-score was expressed in Eq. (16),

16 $$ F = 2\frac{precision \cdot recall}{precision + recall} $$

Given that both recall and precision are evenly weighted, the F-score value lies between 0 and 1, and the recall, precision, F-score should be all ideally close to 1.

#### Distance (D)

To assess the accuracy of the boundary, a curve-based evaluation is performed. Let *C*_*e*_ be the boundary identified by the ophthalmologist and *C*_*m*_ be the boundary achieved by the proposed algorithm. The distance (*D*) which was computed in pixels between two curves was expressed as Eq. (17),

17 $$ D = \frac{1}{n}\sum\limits_{\theta = 1}^{{\theta_{n} }} {\left| {d_{e}^{\theta } - d_{m}^{\theta } } \right|} , $$

Here, *n* is the number of angles, the distance from centroid of *C*_*e*_ to the points on *C*_*e*_ in direction of *θ* was defined as $d_{e}^{\theta } ,$ similar with the definition of $d_{m}^{\theta } .$ D should be close to 0 for an accurate algorithm.

#### Vertical CDR

**Fig. 4:**
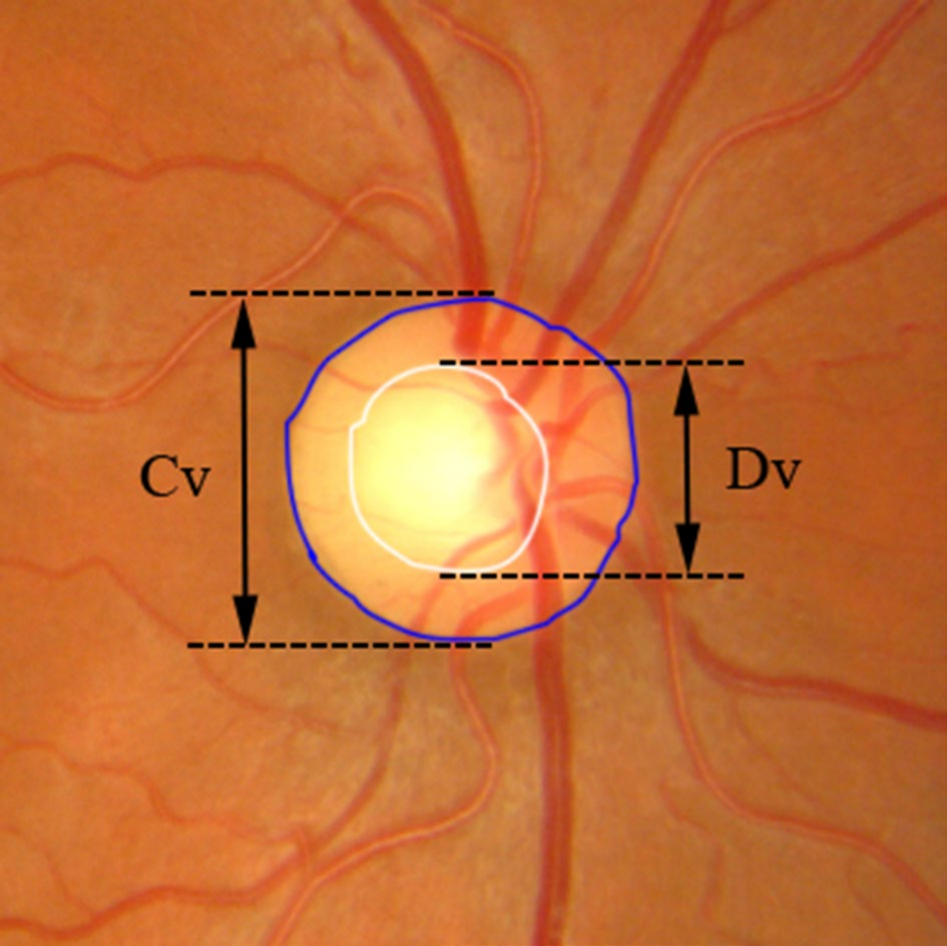
Illustration of vertical CDR measurement criteria

To estimate the vertical CDR, the optic disc must be segmented ahead. Compared to optic cup, optic disc segmentation is relatively easier. In this study, to ensure the accuracy of the CDR and evaluate the effectiveness of the proposed algorithm, the optic disc was manually delineated by experts. The vertical CDR was calculated by Eq. (18) proposed by Gloster et al. [34],

18 $$ CDR = {{C_{V} } \mathord{\left/ {\vphantom {{C_{V} } {D_{V} }}} \right. \kern-0pt} {D_{V} }} $$

where *C*_*V*_ represented the vertical diameter of optic cup, and *D*_*V*_ represented the vertical diameter of optic disc. *C*_*V*_ and *D*_*V*_ were determined by the distances from the top to the bottom of these diameters, as shown in Fig. 4. In clinic, the risk of suffering glaucoma increases with the value of CDR.

## Results

A total of 94 digital fundus images, including 32 glaucoma images, were experimented using the proposed algorithm. The algorithm was individually evaluated against manual segmentation results by expert-1 and expert-2, and also against the average expert marking called expert-X. For comparison, the results obtained by two other known methods of Joshi et al. [17] and Liu et al. [21], were also presented in evaluation of F-score and distance.

**Fig. 5:**
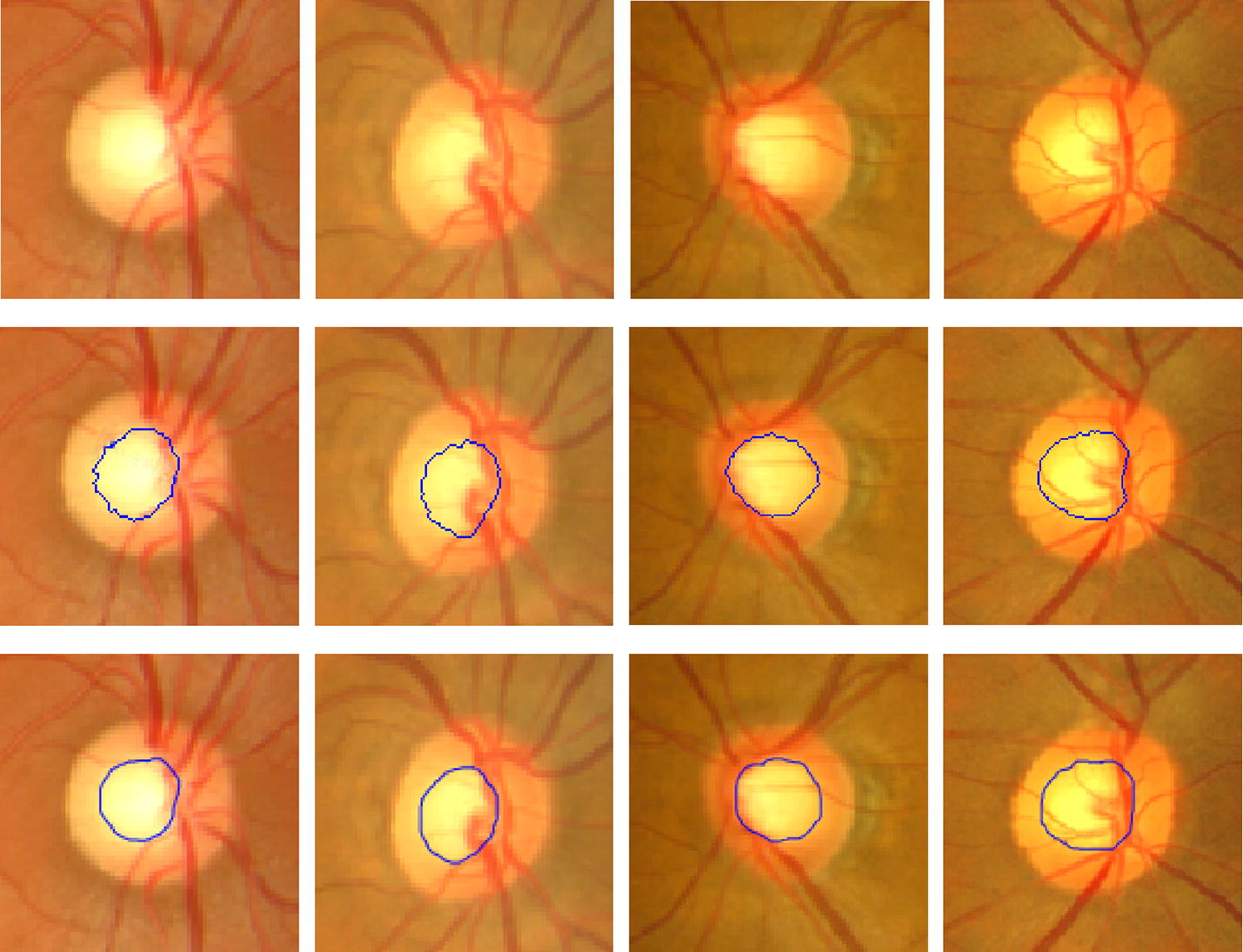
The optic cup segmentation results. From top to bottom are original images, results achieved by our proposed method and by experts respectively

**Fig. 6:**
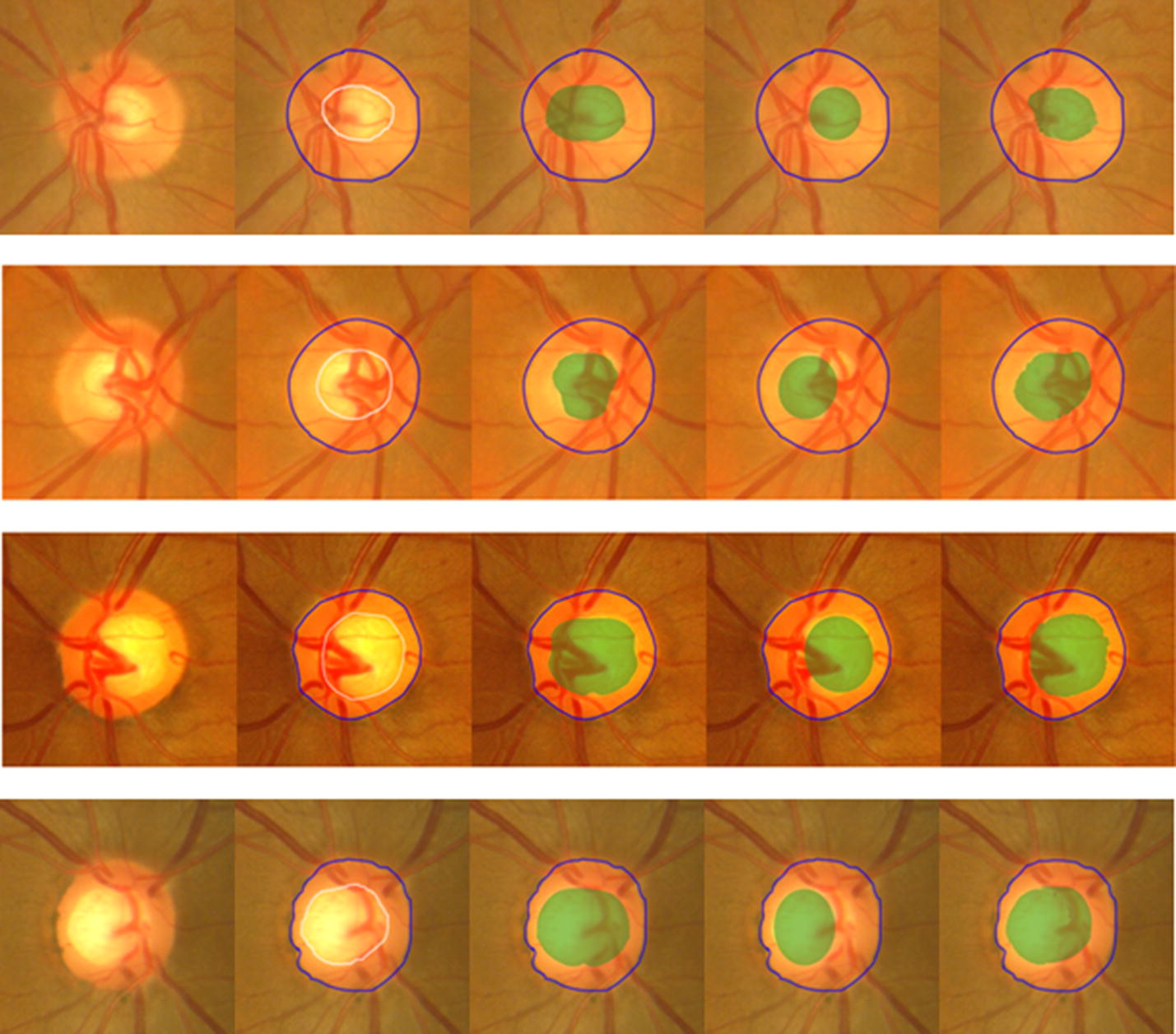
Illustration of comparisons among the segmentation results. From left to right are original images, results achieved by experts, by Joshi et al., by Liu et al. and our proposed method

**Fig. 7:**
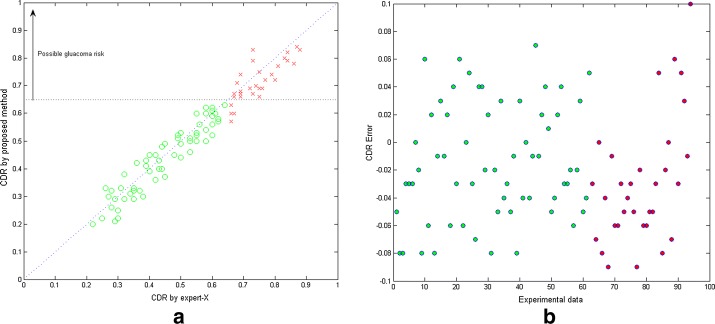
Comparison results of CDR achieved by experts and the proposed method. **a** CDR values achieved by experts vs. by proposed method, **b** errors between the results by experts and the proposed algorithm

The optic cup segmentation results were shown in Fig. 5. In order to compare with the other above mentioned methods, more examples were shown in Fig. 6. The evaluation results of F-score and distance were shown in Tables 1 and 2. The values of CDR achieved by experts and the proposed method were also compared shown in Fig. 7. The statistical results of CDR for normal and glaucoma data were listed in Table 3.

**Table 1:** F-score acquired by different methods

Expert	Joshi et al. [17]	Liu et al. [21]	Proposed
Expert-1	0.6125 ± 0.1139	0.7010 ± 0.0901	0.7955 ± 0.0724
Expert-2	0.6235 ± 0.0999	0.7245 ± 0.1032	0.7780 ± 0.0794

**Table 2:** Boundary distance in radial direction acquired by different methods (in pixels)

Expert	Joshi et al. [17]	Liu et al. [21]	Proposed
Expert-1	22.82 ± 5.00	16.78 ± 3.95	11.42 ± 3.61
Expert-2	20.82 ± 4.08	15.78 ± 4.40	12.32 ± 3.71

**Table 3:** Average CDR values for normal and glaucoma

Method	Normal	Glaucoma
Proposed	0.4369 ± 0.1193	0.7156 ± 0.0698
Expert	0.4516 ± 0.1176	0.7444 ± 0.0666

**Fig. 8:**
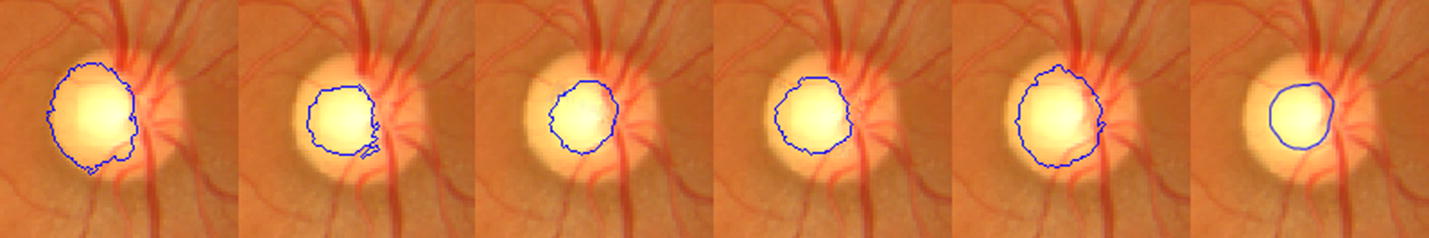
Comparison results with different SE size. From left to right are optic cup segmentation results acquired without morphometric operation, with size of 2 × 2 pixels, 5 × 5 pixels, 10 × 10 pixels, 15 × 15 pixels and by the experts

**Fig. 9:**
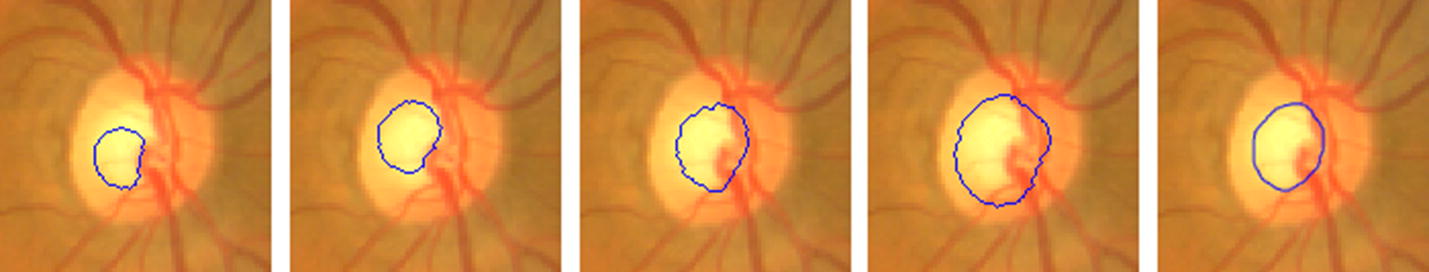
Comparison results with different window size of median filtering. From left to right are optic cup segmentation results acquired with size of 5 × 5 pixels, 7 × 7 pixels, 9 × 9 pixels, 11 × 11 pixels and by the experts

The selections of parameters used in the algorithm were also evaluated. For the SE used in morphometric operations of preprocessing, the size from 2 × 2 pixels to 15 × 15 pixels have been experimented. Representative results were shown in Fig. 8 and Table 4. Representative results of median filtering (window size is from 3 × 3 pixels to 15 × 15 pixels, interval of 2 × 2 pixels) were shown in Fig. 9 and Table 5.

**Table 4:** The precisions of optic cup segmentation with different size of structuring element used in the morphometric operations

Image type	2 × 2 pixels	5 × 5 pixels	10 × 10 pixels	15 × 15 pixels
Normal	0.7950 ± 0.0600	0.8914 ± 0.0300	0.6964 ± 0.0600	0.5900 ± 0.1600
Glaucoma	0.5753 ± 0.2000	0.7218 ± 0.0400	0.4415 ± 0.2100	0.2519 ± 0.1100

**Table 5:** The precisions of optic cup segmentation with different size of median filtering

Image type	5 × 5 pixels	7 × 7 pixels	9 × 9 pixels	11 × 11 pixels
Normal	0.5250 ± 0.0600	0.7114 ± 0.0300	0.8914 ± 0.0300	0.6900 ± 0.1600
Glaucoma	0.3951 ± 0.2000	0.5618 ± 0.0400	0.7218 ± 0.0400	0.5219 ± 0.1100

From the results of Figs. 5 and 6, the proposed algorithm achieved the satisfied segmentation results which were better than the methods proposed by Joshi et al. [17] and Liu et al. [21]. Although both of them also considered the influence caused by blood vessels and proposed the corresponding ways trying to decrease the influence, our proposed method showed competitive to overcome the difficulty in areas with a lot of blood vessels. In addition, both the maximum F-score and minimum distance of our proposed method were acquired. That is, the accuracy of the proposed method is better than the other two. Furthermore, the standard deviations of F-score and distance were also lower which showed the robustness of the proposed method.

From results of Fig. 8 and Table 4, the size of SE was suggested to be selected as 5 × 5. From results of Fig. 9 and Table 5, the window size of median filtering was suggested to be selected as 9 × 9.

In order to evaluate whether our proposed method is feasible and comparable to the most recent published optic cup segmentation algorithms [35–37], also 39 glaucoma images from the public database RIM-ONE were experimented. F-score and CDR acquired by our proposed method and these referenced algorithms were listed in Table 6. From the results, F-score acquired by our proposed method is acceptable and the corresponding CDR is more close to the manual results by experts.

**Table 6:** Comparison with the recent algorithms based on public database RIM-ONE

Criteria	Haleem et al. [35]	Al-Bander et al. [36]	Bechar et al. [37]	Proposed	Expert
F-score	–	0.6903	0.8643	0.8127	–
CDR	0.60 ± 0.17	–	–	062 ± 0.17	0.66 ± 0.18

## Discussion

The proposed algorithm and the other two above mentioned methods used for comparison all put forward the solutions aimed at reducing optic cup segmentation errors caused by blood vessels. Given the figures listed in results, it can be concluded that the proposed method will give rise to higher F and lower D, which was hence better in optic cup segmentation. Joshi et al. [17] imposed the expected symmetry of cup region in nasal and temporal side after threshold processing to recover the vessel errors. A vertical axis of symmetry passing through optic disc center was considered and nasal region was obtained by mirroring the temporal region. Therefore, the segmentation results were decided completely by the segmentation quality of temporal region. However, most of the cups are not symmetrical about the vertical axis passing through optic disc center. What is more, as the acquisition conditions like photographic angle changes, the shapes of both sides are various which also causes the uncertainty of the relative size on both sides, leading to under-segmentation or over-segmentation of nasal side after the operation of symmetry. Liu et al. [21] applied ellipse fitting to redraw the cup boundary for weakening vessel influence in the segmentation process. Nevertheless, the ellipse fitting was implemented after employing combinative algorithm of level set and color intensity thresholds which didn’t take the vessel into account because of its low intensity. Thus only the minor remission of the errors caused by vessels could be reached after fitting, serious under-segmentation still cannot be avoided. By contrast, the proposed scheme firstly fills the blood vessels by an improved BSCB model, making the whole cup area more uniform for the following segmentation,then the optic cup is determined through the LCV model approach which can capture the unsharp boundary of cup, reducing under-segmentation or over-segmentation to a great extent compared with other methods.

For the CDR evaluation in Table 3, the CDR values by the proposed algorithm are little smaller than those by expert, which indicates that the cup region determined by the proposed method was little smaller toward upper cup edge and lower cup edge than that determined by expert. The CDR values are little too small caused the misdiagnosis of four patients with glaucoma as normal in the experiment. The reason is that, for the dense vascular area near the cup edge, the grayscale is close to that of the neuroretinal rim after inpainting, leading to little local insufficient segmentation. But the difference of CDR between the proposed method and expert is small, thus the CDR was in the range of normal.

To sum up, we have proposed a novel automatic optic cup segmentation algorithm based on inpainting of blood vessels in this study. Compared with other techniques, the proposed methodology has several advantages.

1. The proposed algorithm repaired the vascular regions by using an improved BSCB model, reducing the errors caused by blood vessels to a large degree and improving the segmentation accuracy;
2. In LCV model, the centroid point located in area with higher intensity was selected as the starting point, which realized the automatic selection of the seed point. In addition, the loose selection of circular radius make the initial contour easily be automatically selected.
3. The proposed algorithm realized fully automatic segmentation with high accuracy, which reduced the human intervention of the traditional semi-automatic method.

The proposed method also has some limitations. First, the region of optic cup segmented by the proposed method was slightly smaller than that identified by experts, especially in the dense vascular area. It may be because parts of the image intensities near the edge of optic cup are close to these of the neuroretinal rim after inpainting. Accordingly, local insufficient segmentation will be happened. Second, the robustness of the algorithm is expected to improve. Since the used experimental data were collected from the same type of camera, more data from different type of camera should be tested and the robustness of the algorithm will be improved.

## Conclusion

This study proposed a novel optic cup segmentation method based on inpainting of blood vessels using an improved BSCB model. The proposed algorithm realized fully automatic segmentation, which captured the optic cup boundary more accurately compared with other previous methods also dedicating to reduce the influence of blood vessels. Future work is needed to experimented more samples acquired from different type of camera and also developed more robust algorithms.

## References

[1] Weinreb RN, Aung T, Medeiros FA (2014). The pathophysiology and treatment of glaucoma: a review. JAMA.

[2] Lee S, Young M, Sarunic MV (2011). End-to-end pipeline for spectral domain optical coherence tomography and morphometric analysis of human optic nerve head. J Med Biol Eng.

[3] Chen SLJ (2011). Detection of the optic disc on retinal fluorescein angiograms. J Med Biol Eng.

[4] Shen SY, Wong TY, Foster PJ (2008). The prevalence and types of glaucoma in Malay people: the Singapore Malay Eye Study. Invest Ophthalmol Vis Sci.

[5] Cheng JW, Cheng SW, Ma XY (2013). The prevalence of primary glaucoma in mainland China: a systematic review and meta-analysis. J Glaucoma.

[6] Dirani M, Crowston JG, Taylor PS (2011). Economic impact of primary open-angle glaucoma in Australia. Clin Exp Ophthalmol.

[7] Michelson G, Wärntges S, Hornegger J (2008). The papilla as screening parameter for early diagnosis of glaucoma. Dtsch Arztebl Int.

[8] Hatanaka Yuji, Noudo Atsushi, Muramatsu Chisako, Sawada Akira, Hara Takeshi, Yamamoto Tetsuya, Fujita Hiroshi (2010). Automatic Measurement of Vertical Cup-to-Disc Ratio on Retinal Fundus Images. Lecture Notes in Computer Science.

[9] Xu Y, Hu M, Jia X, et al. Computer-aided diagnosis of glaucoma using fundus images. In: Proceedings of the 2014 international conference on mechatronics, electronic, industrial and control engineering; 2014.

[10] Fujita H, Uchiyama Y, Nakagawa T, et al. CAD on brain, fundus, and breast images. In: International conference on medical imaging and informatics. Berlin: Springer; 2008. p. 358–66.

[11] Yin F, Liu J, Wong DWK, et al. Automated segmentation of optic disc and optic cup in fundus images for glaucoma diagnosis. In: IEEE CBMS 2012: 25th international symposium on computer-based medical systems (CBMS). New York: IEEE; 2012. p. 1–6.

[12] Zhang Z, Srivastava R, Liu H (2014). A survey on computer aided diagnosis for ocular diseases. BMC Med Inform Decis Mak.

[13] Bock R, Meier J, Nyúl LG (2010). Glaucoma risk index: automated glaucoma detection from color fundus images. Med Image Anal.

[14] Tangelder GJM, Reus NJ, Lemij HG (2006). Estimating the clinical usefulness of optic disc biometry for detecting glaucomatous change over time. Eye.

[15] Nayak J, Acharya R, Bhat PS (2009). Automated diagnosis of glaucoma using digital fundus images. J Med Syst.

[16] Babu TRG, Shenbagadevi S (2011). Automatic detection of glaucoma using fundus image. Eur J Sci Res.

[17] Joshi GD, Sivaswamy J, Karan K, et al. Optic disk and cup boundary detection using regional information. In: 2010 IEEE international symposium on biomedical imaging: from nano to macro. New York: IEEE; 2010. p. 948–51.

[18] Xiao D, Lock J, Manresa JM, et al. Region-based multi-step optic disk and cup segmentation from color fundus image. In: SPIE medical imaging. International Society for Optics and Photonics; 2013. p. 86702H–8.

[19] Madhusudhan M, Malay N, Nirmala SR, et al. Image processing techniques for glaucoma detection. In: International conference on advances in computing and communications. Berlin: Springer; 2011. p. 365–73.

[20] Joshi GD, Sivaswamy J, Krishnadas SR (2011). Optic disk and cup segmentation from monocular color retinal images for glaucoma assessment. IEEE Trans Med Imaging.

[21] Liu J, Wong DWK, Lim JH, et al. ARGALI: an automatic cup-to-disc ratio measurement system for glaucoma detection and analysis framework. In: SPIE medical imaging. Bellingham: International Society for Optics and Photonics; 2009: 72603K–8.

[22] Liu J, Wong DWK, Lim JH, et al. ARGALI: an automatic cup-to-disc ratio measurement system for glaucoma analysis using level-set image processing. In: 13th International conference on biomedical engineering. Berlin: Springer; 2009. p. 559–62.

[23] Zhang Z, Liu J, Cherian NS, et al. Convex hull based neuro-retinal optic cup ellipse optimization in glaucoma diagnosis. In: 2009 annual international conference of the IEEE engineering in medicine and biology society. New York: IEEE; 2009. p. 1441–4. 10.1109/IEMBS.2009.5332913 19963748

[24] Wong DWK, Liu J, Lim JH, et al. Automated detection of kinks from blood vessels for optic cup segmentation in retinal images. In: SPIE medical imaging. Bellingham: International Society for Optics and Photonics; 2009. p. 72601J–8.

[25] Cheng J, Liu J, Xu Y (2013). Superpixel classification based optic disc and optic cup segmentation for glaucoma screening. IEEE Trans Med Imaging.

[26] Cheng Jun, Liu Jiang, Tao Dacheng, Yin Fengshou, Wong Damon Wing Kee, Xu Yanwu, Wong Tien Yin (2013). Superpixel Classification Based Optic Cup Segmentation. Medical Image Computing and Computer-Assisted Intervention – MICCAI 2013.

[27] Xu Y, Liu J, Cheng J, et al. Efficient optic cup localization based on superpixel classification for glaucoma diagnosis in digital fundus images. In: 2012 21st international conference on pattern recognition (ICPR). IEEE; 2012. p. 49–52.

[28] Fumero F, Alayón S, Sanchez JL, et al. RIM-ONE: an open retinal image database for optic nerve evaluation. In: 2011 24th international symposium on computer-based medical systems (CBMS). IEEE; 2011. p. 1–6.

[29] Dougherty ER, Lotufo RA, The International Society for Optical Engineering SPIE (2003). Hands-on morphological image processing.

[30] Bertalmio M, Sapiro G, Caselles V, et al. Image inpainting. In: Proceedings of the 27th annual conference on computer graphics and interactive techniques. New York: ACM Press/Addison-Wesley Publishing Co.; 2000. p. 417–24.

[31] Perona P, Malik J (1990). Scale-space and edge detection using anisotropic diffusion. IEEE Trans Pattern Anal Mach Intell.

[32] Catté F, Lions PL, Morel JM (1992). Image selective smoothing and edge detection by nonlinear diffusion. SIAM J Numer Anal.

[33] Wang XF, Huang DS, Xu H (2010). An efficient local Chan–Vese model for image segmentation. Pattern Recogn.

[34] Gloster J, Parry DG (1974). Use of photographs for measuring cupping in the optic disc. Br J Ophthalmol.

[35] Haleem MS, Han L, van Hemert J (2018). A novel adaptive deformable model for automated optic disc and cup segmentation to aid glaucoma diagnosis. J Med Syst.

[36] Al-Bander B, Williams BM, Al-Nuaimy W (2018). Dense fully convolutional segmentation of the optic disc and cup in colour fundus for glaucoma diagnosis. Symmetry.

[37] Bechar MEA, Settouti N, Barra V (2018). Semi-supervised superpixel classification for medical images segmentation: application to detection of glaucoma disease. Multidimens Syst Signal Process.

